# A *Meloidogyne graminicola* Pectate Lyase Is Involved in Virulence and Activation of Host Defense Responses

**DOI:** 10.3389/fpls.2021.651627

**Published:** 2021-03-22

**Authors:** Jiansong Chen, Zhiwen Li, Borong Lin, Jinling Liao, Kan Zhuo

**Affiliations:** ^1^Guangdong Laboratory of Lingnan Modern Agriculture, Guangzhou, China; ^2^Laboratory of Plant Nematology, South China Agricultural University, Guangzhou, China; ^3^Guangdong Eco-Engineering Polytechnic, Guangzhou, China

**Keywords:** *Meloidogyne graminicola*, pectate lyase, enzymatic activity, plant immunity elicitation, RNAi

## Abstract

Plant-parasitic nematodes secrete an array of cell-wall-degrading enzymes to overcome the physical barrier formed by the plant cell wall. Here, we describe a novel pectate lyase gene *Mg-PEL1* from *M. graminicola*. Quantitative real-time PCR assay showed that the highest transcriptional expression level of *Mg-PEL1* occurred in pre-parasitic second-stage juveniles, and it was still detected during the early parasitic stage. Using *in situ* hybridization, we showed that Mg-PEL1 was expressed exclusively within the subventral esophageal gland cells of *M. graminicola.* The yeast signal sequence trap system revealed that it possessed an N-terminal signal peptide with secretion function. Recombinant Mg-PEL1 exhibited hydrolytic activity toward polygalacturonic acid. Rice plants expressing RNA interference vectors targeting *Mg-PEL1* showed an increased resistance to *M. graminicola*. In addition, using an *Agrobacterium*-mediated transient expression system and plant immune response assays, we demonstrated that the cell wall localization of Mg-PEL1 was required for the activation of plant defense responses, including programmed plant cell death, reactive oxygen species (ROS) accumulation and expression of defense-related genes. Taken together, our results indicated that Mg-PEL1 could enhance the pathogenicity of *M. graminicola* and induce plant immune responses during nematode invasion into plants or migration in plants. This provides a new insight into the function of pectate lyases in plants-nematodes interaction.

## Introduction

In nature, plants live in complex environments where they are continually exposed to various beneficial or harmful microbes (Rodriguez et al., 2019). To survive, plants have evolved a sophisticated immune system against pathogenic microbes. It is now clear that plants have two layers of the immune system. The first line of immunity relies on the perception of pathogen-associated molecular patterns (PAMPs) by pattern recognition receptors (PRRs), leading to PAMP-triggered immunity (PTI) (Jones and Dangl, 2006). Such a recognition triggers a cascade of immune events, including calcium influx, the burst of reactive oxygen species (ROS), deposition of callose and expression of defense-related genes (Couto and Zipfel, 2016). For a successful infection, pathogens need to subvert PTI. Adapted pathogens employ numerous effector proteins, which are secreted into plant cells to evade or interfere with PTI, resulting in effector-triggered susceptibility (Win et al., 2012). In the second line of plant immunity, plants make use of nucleotide-binding leucine-rich repeat (NB-LRR) receptor proteins to recognize pathogen effectors, resulting in effector-triggered immunity (ETI) (Jones and Dangl, 2006). In general, ETI leads to a strong defense response associated with a local hypersensitive cell death response (HR). HR-based cell death is believed to effectively limit biotrophic pathogens spread within plant cells (Wilkinson et al., 2019). Besides pathogen-derived PAMPs, the plant innate immune system can recognize some endogenous molecules of the host once the molecules are released into the extracellular environment during tissue disruption (Hou et al., 2019). These host-derived molecules are referred to as damage-associated molecular patterns (DAMPs), which are endogenous indicators of damage and can act as early and general activators of the plant immune system (Choi and Klessig, 2016; Quintana-rodriguez et al., 2018).

The plant cell wall is considered as the first barrier that protects against invading pathogens (Soitamo et al., 2011). The primary plant cell wall is composed of cellulose, hemicellulose, and pectin (Lerouxel et al., 2006). Correspondingly, pathogens including phytopathogenic bacteria, fungi and plant-parasitic nematodes (PPNs) have also evolved numerous kinds of cell-wall-degrading enzymes (CWDEs) to destroy plant cell wall-based defense structure during infection (Kikot et al., 2009; Kubicek et al., 2014). Among the components of a plant cell wall, pectin is the most complex family of acidic polysaccharides, which are classified into three groups: homogalacturonan (α-D-galacturonan or polygalacturonate, PG), rhamnogalacturonan I (RGI), and rhamnogalacturonan II (RGII) (Lerouxel et al., 2006). Therefore, complete pectin degradation requires various pectinases, including pectin lyases, pectate disaccharide-lyases, and pectate lyases (Pereira et al., 2014). Of these, pectate lyase is an important hydrolytic enzyme that cleaves α-1,4-polygalacturonic acid randomly and releasing pectin products, that may induce immune responses within plants (Gui et al., 2017). In the past several decades, some pectate lyases from plant pathogens were found to contribute to virulence and inducing host defense responses (Hugouvieux-Cotte-Pattat et al., 2014), such as endopolygalacturonase 1 T4BcPG1 of *Botrytis cinerea* fungus, pectate lyases from *Erwinia carotovora* bacterium and the oomycete pathogen *Phytophthora capsici* (Wegener et al., 1996; Poinssot et al., 2003; Fu et al., 2015).

PPNs are the only known animal with the ability to produce endogenous pectate lyases (Popeijus et al., 2000). Intriguingly, PPN pectate lyases are believed to originate from bacterial or fungal sources by the horizontal gene transfer (Vanholme et al., 2007). They also play a role in cleaving α-1,4-polygalacturonic acid of pectin, loosen plant cell wall and facilitate penetration and migration of PPNs (Doyle and Lambert, 2002). The first PPN-derived pectate lyase, named Gr-PEL-1, was isolated from the potato cyst nematode *Globodera rostochiensis* (Popeijus et al., 2000). So far pectate lyases have been found in many economically important PPNs, such as *Meloidogyne incognita*, *M. javanica*, *M. enterolobii*, *Heterodera glycines*, *H. schachtii*, *Bursaphelenchus xylophilus*, and *Aphelenchus avenae* (Doyle and Lambert, 2002; Huang et al., 2005; Kikuchi et al., 2006; Kudla et al., 2007; Vanholme et al., 2007; Karim et al., 2009; Zhuo et al., 2011). Some pectate lyases have been demonstrated to fulfill the virulence function and promote nematode infection (Vanholme et al., 2007; Peng et al., 2016). However, whether PPN pectate lyases can induce host immunity is unknown. In this study, a pectate lyase gene *Mg-PEL1* was identified from *M. graminicola*. Here, we showed that the pectate lyase Mg-PEL1 not only plays a role in *M. graminicola* infection as an effector, but also induces plant immune responses.

## Materials and Methods

### Nematode Culture and Collection

*M. graminicola* population was isolated from cultivated rice in Hainan, China, purified using a single egg mass and maintained on rice (*Oryza sativa* cv. “Nipponbare”) roots at 28°C in sterilized sand and soil mixture (3:1). Pre-parasitic second-stage juveniles (pre-J2s) were collected as described previously (Huang et al., 2020).

### Gene Cloning and Sequence Analysis

Total RNA and DNA were extracted from approximately 10,000 pre-J2s using RNAprep Pure Micro Kit (TianGen Biotech, Beijing, China) and TIANamp Micro DNA Kit (TianGen Biotech, Beijing, China), respectively. The first-stand cDNA was synthesized using the TransScript One-Step gDNA Removal and cDNA Synthesis Super Mix (TransGen Biotech, Beijing, China) according to the manufacturer’s instructions. *Mg-PEL1* full-length cDNA sequence and genomic sequence were amplified by the primers Mg-PEL1_F and Mg-PEL1_R, which were designed according to a pectate lyase gene from *M. graminicola* transcription data (Haegeman et al., 2013) and the other pectate lyase gene (AID59201) deposited in GenBank. The PCR conditions were as follows: pre-denaturation at 94°C for 4 min, and 30 cycles of denaturation at 98°C for 10 s, annealing at 55°C for 30 s and polymerization at 68°C for 1 min. Primers used in this study were synthesized by Guangzhou IGE biotechnology, Ltd. (IGE biotechnology, Guangzhou, China) and listed in Supplementary Table 1.

The alignment of pectate lyase sequences from different PPNs was produced by ClustalW^1^ and visualized by Boxshade software^2^. Signal peptide (SP) and transmembrane domain of Mg-PEL1 were predicted using SignalP 5.0^3^ and TMHMM Server v.2.0^4^. The phylogeny tree was reconstructed using Neighbor-Joining (NJ) method by MEGA 7 software with a bootstrap test (1000 replicates) (Kumar et al., 2016). Protein molecular weight was predicted using DNAstar software.

### *In situ* Hybridization and Developmental Expression Analysis

A 632-bp fragment of *Mg-PEL1* from 46 to 677 was selected and amplified using the primer pairs Mg-insitu_F/Mg-insitu_R. Then, the fragment sequence of *Mg-PEL1^46–677^* was used to synthesize digoxigenin (DIG)-labeled cDNA sense and antisense probes by the PCR DIG Probe Synthesis Kit (Roche, Rotkreuz, Switzerland) according to the manufacturer’s instructions. Approximately 10,000 pre-J2s of *M. graminicola* were prepared for fixation and hybridization as described previously (de Boer et al., 1998; Haegeman et al., 2013), and the signals were visualized and captured using Nikon ECLIPSE Ni microscope (Nikon, Tokyo, Japan).

Transcript levels of *Mg-PEL1* were measured at different developmental stages of *M. graminicola* by quantitative real-time PCR (qRT-PCR). Total RNA isolation and first-strand cDNA synthesis were performed as described above. qRT-PCR was performed using the primer pairs Mg-PEL1_qPCR_F/Mg-PEL1_qPCR_R and Mgactin2-F/Mgactin2-R for amplifying the gene *Mg-PEL1* and the endogenous reference gene *Mgactin-2* (Haegeman et al., 2013). qRT-PCR was performed using SYBR Premix Ex Taq II (Tli RNaseH Plus) (Takara, Tokyo, Japan) on a Thermal Cycler Dice Real-Time System (Takara, Tokyo, Japan). Fold changes in gene expression were determined using the 2^–△^^△^^*CT*^ method (Livak and Schmittgen, 2001). Three independent experiments with three technical replicates in each experiment were performed.

### Yeast Signal Sequence Trap System

The vector pSUC2 contains a truncated invertase gene, which lacks Methionine (Met) and SP sequence. The SP sequence of *Mg-PEL1* was cloned into pSUC2 to generate pSUC2:Mg-PEL1(SP)-Invertase. And the SP of *Phytophthora sojae* RXLR effector Avr1b was also cloned into pSUC2 to generate pSUC2:Avr1b(SP)-Invertase as a positive control. This SP has been proven to have secretion function in yeast (Dou et al., 2008). pSUC2: Mg-PEL1(SP)-Invertase and pSUC2:Avr1b (SP)-Invertase were transformed into the yeast strain YTK12 by the lithium acetate method (Gietz and Schiestl, 2007; Yin et al., 2018). The yeast strain YTK12 and YTK12 carrying pSUC2 empty vector were used as negative controls. YTK12 and YTK12 containing pSUC2-derived plasmids were grown on the medium CMD-W (0.67% yeast N base without amino acids, 0.075% W dropout supplement, 2% sucrose, 0.1% glucose, and 2% agar) to detect the expression of pSUC2-derived plasmids. Yeast transformants were grown on the YPRAA plate [1% yeast extract, 2% peptone, 2% raffinose, and antimycin A (2 mg/ml)] to detect invertase secretion. Enzymic activity of invertase was further confirmed by the reduction of the dye 2, 3, 5-triphenyltetrazolium chloride (TTC) to the insoluble red-colored triphenylformazan. The detailed procedure was described previously (Yin et al., 2018).

### Western Blot

Total proteins were extracted using RIPA lysis buffer (2% SDS, 80 mM Tris/HCl, pH 6.8, 10% glycerol, 0.002% bromophenol blue, 5% β-mercaptoethanol, and complete protease inhibitor cocktail). Sample proteins were denatured and separated on a 12% SDS-PAGE gel, and transferred to a nitrocellulose membrane (PALL, Washington, NY, United States). After blocking with 5% (w/v) non-fat milk for 1 h at room temperature, the membranes were incubated with a primary mouse anti-Flag, anti-Strep or anti-GFP antibody (1:5,000 dilution) (Roche, Rotkreuz, Switzerland) in blocking solution for 2 h. Then membranes were incubated with an anti-mouse horseradish peroxidase-conjugated secondary antibody at a 1:5,000 dilution (Biosynthesis Biotechnology Co., Beijing, China). The proteins were visualized using the Immobilon Western Chemiluminescent system (Merck, Darmstadt, Germany), and Ponceau S staining was used to show equal loading (Goldman et al., 2016).

### Enzymatic Activity Assay

Extracellular enzyme activity of pectate lyase was monitored using a titrimetric stop reaction method (Starr et al., 1977). In brief, the full-length sequence of *Mg-PEL1* was cloned into the yeast expression vector pSUC2 to generate a pSUC2: Mg-PEL1: Strep fusion construct. The Mg-PEL1: Strep fusion protein was concentrated by immunoprecipitation with Strep beads. Subsequently, Coomassie brilliant blue staining and western blots were used to confirm the expression of Mg-PEL1: Strep fusion protein in supernatant solution. The purified fusion proteins were mixed with 0.5% (wt/vol) polygalacturonic acid (PGA) (Sigma-Aldrich, MO, United States) solution (0.05 M Tris-HCl, 1 mM Ca^2+^, pH 8.5) and inoculated at 25°C for 0 h and 16 h, respectively. Then, 0.5 ml I_2_ (100 mM) solution and 0.1 ml Na_2_CO_3_ (1 M) were added to the reaction mixture and incubated in the dark for 20 min. The mixtures were then acidified by adding 0.2 ml of H_2_SO_4_ (1 M). The free iodine was titrated with continuous stirring against 50 mM Na_2_S_2_O_3_ using 1.0% (w/v) starch as an indicator.

### Construction of RNAi Expression Vector and Plant Transformation

A 336 bp fragment of *Mg-PEL1* from 368 to 703 bp was selected as the template for RNAi-mediated silencing in rice. CaMV35S-promotor of the pCAMBIA1305.1 vector was replaced with the maize ubiquitin promoter to generate the binary vector pUbi (Chen et al., 2017). The complete RNAi hairpin structure containing *Mg-PEL1*^368–703^ (both sense and antisense orientation, separated by a β-glucuronidase (GUS) intron) under the control of maize ubiquitin promoter (Ubi) was transformed into *A. tumefaciens* strain EHA105 by electroporation. Rice callus induction and co-cultivation with *A. tumefaciens* were performed as described previously (Kim et al., 2017). The transgenic seedlings were screened on an N6 medium containing 50 mg/L hygromycin. Semi-quantitative reverse-transcriptase polymerase chain reaction (Semi-qRT-PCR) was used to measure the expression level of RNAi construct in each transgenic rice line using the GUS intron as a target fragment. To evaluate the expression level of *Mg-PEL1* in *M. graminicola* parasitizing rice lines expressing the RNAi expression vector, total RNA was isolated from root segments containing nematodes at 3 d after nematode inoculation. qRT-PCR was performed using the primer pairs Mg-PEL1_qPCR_F/Mg-PEL1_qPCR_R and Mgactin2-F/Mgactin2-R for amplifying the target gene *Mg-PEL1* and the endogenous reference gene *Mgactin-2* of *M. graminicola*, respectively. In addition, two other genes, *Mg-CRT* and *Mg-expansin*, were also evaluated to determine the specificity of *Mg-PEL1* silencing (Chen et al., 2017). All mRNA samples were extracted from three independent samples and quantitative or semi-qRT-PCR were performed with three technical replicates for each sample.

### Plant Material and Infection Assay

Rice plants were grown in a growth chamber at 28°C under 16 h light and 8 h dark conditions in Quartz sand. 2 week-old rice seedlings were inoculated with about 300 pre-J2s. Fifteen days after nematode inoculation, the rice roots were collected, washed and stained by acid fuchsine (Naalden et al., 2018b), and the number of nematodes and adult females were counted. For phenotypic analysis of transgenic rice, 4 week-old rice seedlings were collected, and plant height, root length and root weight were measured. These experiments were performed two times. Statistically significant differences between transgenic lines and wild type (WT) were determined by a Student’s *t*-test.

### Subcellular Localization

The *Mg-PEL1* sequence with or without the native SP was amplified and cloned into pCAMBIA1305.1 vector to generate Mg-PEL1: GFP and Mg-PEL1^–SP^: GFP, respectively. The SP sequence of pathogenesis-related protein 1a (PR1a) (Supplementary Figure 1) was amplified from *N. benthamiana* and fused with Mg-PEL1 lacking the native SP to generate Mg-PEL1^PR1a^: GFP. PR1a is an important defense protein of plants and accumulates predominantly in the apoplastic space (Pecenkova et al., 2017). All constructs were transformed into an *A. tumefaciens* strain GV3101 and transiently expressed in *N. benthamiana* leaves by agroinfiltration. To confirm the apoplastic localization, *N. benthamiana* leaves expressing constructs were infiltrated with 30% glycerol for 20 min to induce plasmolysis (Liu et al., 2016). Subsequently, photographs were taken using an SP5 Leica confocal microscope (Nikon, Tokyo, Japan).

### Plant Immune Response Assay

*N. benthamiana* was grown in a controlled greenhouse at 25°C under 16 h light and 8 h dark conditions for a month. One day before agroinfiltration, plants were transferred into a growth chamber and maintained at 20–25°C, 35–50% humidity, with a 16 h light/8 h dark cycle. *A. tumefaciens* strain GV3101 carrying four constructs Mg-PEL1:Flag, Mg-PEL1^–SP^:Flag, Mg-PEL1^PR1a^:Flag and Flag were transiently expressed in *N. benthamiana* leaves. Images were taken for the cell death symptom analysis at 5 d after infiltration. To detect the accumulation of hydrogen peroxide, four constructs Mg-PEL1:Flag, Mg-PEL1^–SP^:Flag, Mg-PEL1^PR1a^:Flag and Flag were infiltrated into leaves of 4 week-old *N. benthamiana*. Two days after infiltration, *N. benthamiana* leaves were stained by using 3,3’-diaminobenzidine (DAB) solution as described previously (Daudi and O’Brien, 2012). To determine the defense-related gene expression levels, *A. tumefaciens* strains carrying the constructs Mg-PEL1:Flag, Mg-PEL1^–SP^:Flag, Mg-PEL1^PR1a^:Flag and Flag were infiltrated into *N. benthamiana* leaves. Two days after infiltration, the transcript abundance of three representative defense-related genes, *PR-5*, *PAL*, and *NPR1*, was determined by semi-qRT-PCR, and *NbEF1*α (Heese et al., 2007) was used as the reference gene.

## Results

### Sequence Analysis of the *Mg-PEL1* Gene From *M. graminicola*

A 972-bp genomic DNA sequence of *Mg-PEL1* (GenBank accession number MW266124) was amplified from *M. graminicola*. *Mg-PEL1* contains a 795 bp coding region, interrupted by two introns of 140 and 37 bp (Supplementary Figure 2). The complete open read frame (ORF) encodes a 264-amino-acid (aa) protein with a predicted molecular mass of 29.3 kDa. Signal analysis of Mg-PEL1 identified an 18-aa N-terminal SP, and no putative transmembrane domain was predicted.

**FIGURE 1:**
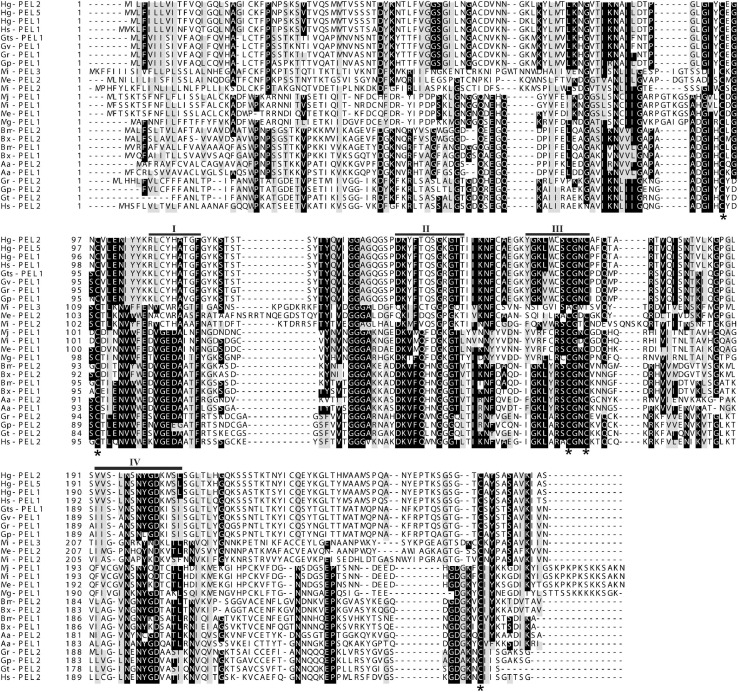
Multiple sequence alignment of the predicted Mg-PEL1 protein with other pectate lyase sequences from plant-parasitic nematodes. Hg-PEL2 (AAM74954), Hg-PEL5 (ADW77536), and Hg-PEL1 (AAK08974) from *Heterodera glycines*; Hs-PEL1 (ABN14273) and Hs-PEL2 (ABN14272) from *H. schachtii*; Gts-PEL1 (AEA08834) and Gt-PEL2 (ACU64859) from *Globodera tabacum*; Gv-PEL1 (AEA08828) from *G. virginiae*; Gr-PEL1 (AAF80746) and Gr-PEL2 (AAM21970) from *G. rostochiensis*; Gp-PEL2 (ACU64845) and Gp-PEL1 (AEA08862) from *G. pallida*; Mi-PEL1 (AAQ09004), Mi-PEL2 (AAQ97032) and Mi-PEL3 (AAW56829) from *Meloidogyne incognita*; Me-PEL1 (ADN87334) and Me-PEL2 (ALB38961) from *M. enterolobii*; Mj-PEL1 (AAL66022) from *M. javanica*; Mg-PEL1 (MW266124) from *M. graminicola*; Bm-PEL1 (BAE48374) and Bm-PEL2 (BAE48375) from *Bursaphelenchus mucronatus*; Bx-PEL1 (BAE48371) and Bx-PEL2 (BAE48372) from *B. xylophilus*; Aa-PEL1 (BAI44499) and Aa-PEL2 (BAI44497) from *Aphelenchus avenae*. Black bars (I–IV) indicate the conserved regions. Black stars indicate the conserved cysteine residues.

A multiple sequence alignment of the deduced amino acid sequence of Mg-PEL1 with pectate lyase sequences from other PPNs is present in Figure 1. Mg-PEL1 contains highly conserved regions that are common to microbial class III pectate lyases (Figure 1) and shows 100% identity with a pectate lyase (AID59201) from *M. graminicola* transcriptome and 99.6% identity (263/264, without insertions/deletions) with the other pectate lyase (KAF7637017) from *M. graminicola* genome. It also shows similarities to three *Meloidogyne* pectate lyases, Me-PEL1 of *M. enterolobii* (ADN87334), Mj-PEL1 of *M. javanica* (AAL66022), Mi-PEL1 of *M. incognita* (AAQ09004), by a protein BLAST search. However, the identities between Mg-PEL1 and these three pectate lyases are only 58.0–59.0%. In addition, Mg-PEL1 displays somewhat similar to Rs-PEL3 of *Radopholus similis* (QIC04078, 50.4% identity) and several pectate lyases from bacteria and fungi, such as *Streptomyces kanasensis* (WP_058944121, 42.0% identity) and *Anthracocystis flocculosa* PF-1 (XP_007881659, 41.1% identity). Between Mg-PEL1 and other PPN pectate lyases, the identities are less than 40.6%. A NJ tree (Supplementary Figure 3) was constructed to examine the relationships among 37 pectate lyases. This phylogenetic tree shows that Mg-PEL1, Me-PEL1, Mj-PEL1, Mi-PEL1, and Rs-PEL3 are placed within a well-supported monophyletic clade; this clade is then sister to the other clade comprising some pectate lyase sequences from bacteria and fungi, but far from other PPN pectate lyase sequences.

### Mg-PEL1 Is Expressed in the Subventral Esophageal Gland Cells of *M. graminicola* and Contains a Signal Peptide With Secretion Function

**FIGURE 2:**
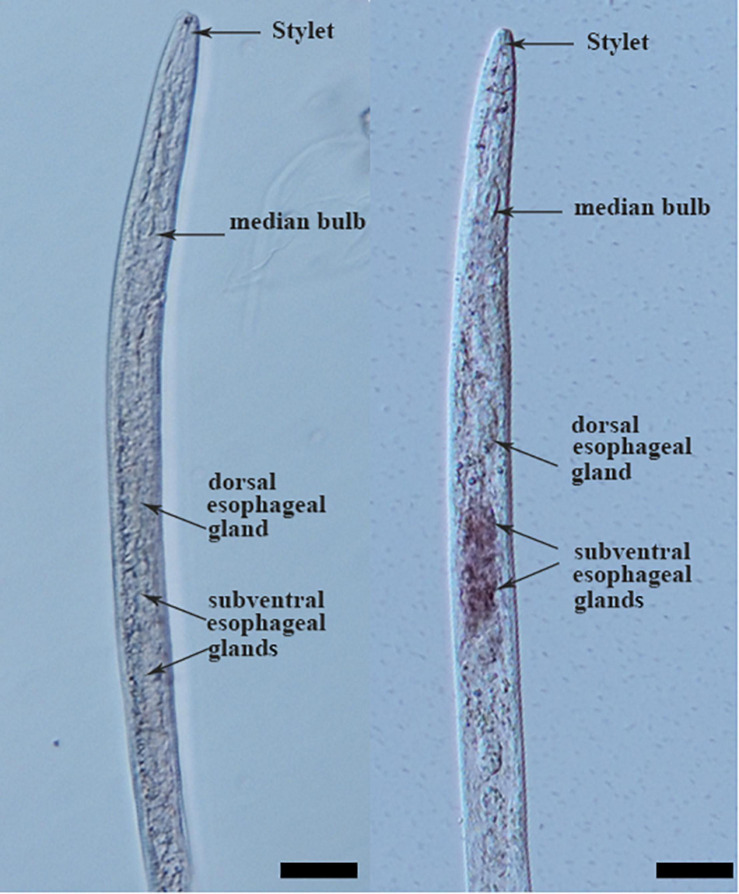
Subventral gland localization of *Mg-PEL1* in the pre-parasitic second stage juveniles of *Meloidogyne graminicola*. Fixed nematodes were hybridized with digoxigenin-labeled *Mg-PEL1* sense cDNA probe (left) and antisense cDNA probe (right). Scale bars, 20 μm.

Tissue localization of *Mg-PEL1* in *M. graminicola* was determined by *in situ* hybridization. Hybridization signal was observed in the subventral esophageal gland cells with the DIG-labeled antisense cDNA probe of *Mg-PEL1* (Figure 2 and Supplementary Figure 4). No hybridization signal was found in the tissue of *M. graminicola* with the sense cDNA probe of *Mg-PEL1* (Figure 2).

**FIGURE 3:**
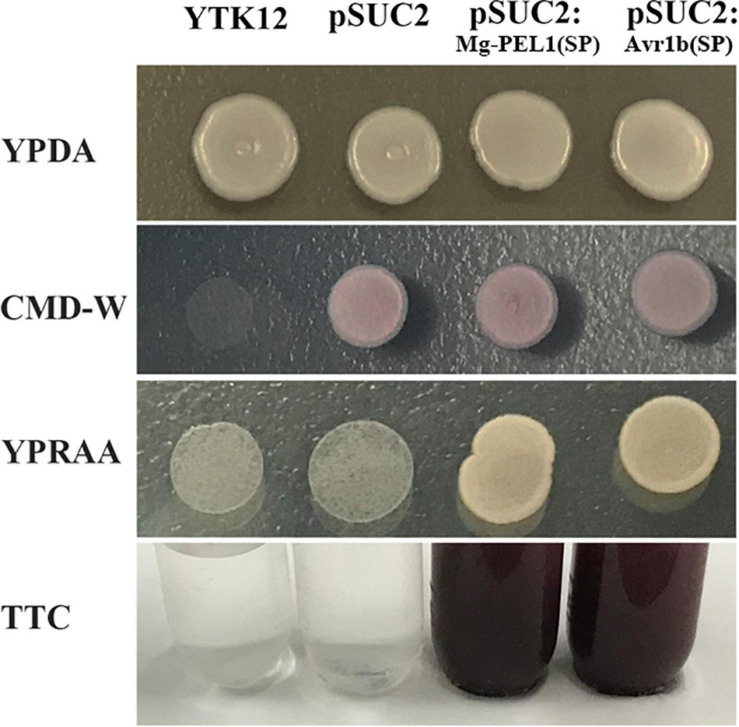
Secretion function analysis of Mg-PEL1 signal peptide. The predicted signal peptide of Mg-PEL1 was cloned into the yeast vector pSUC2 to generate pSUC2:Mg-PEL1(SP)-invertase construct, and pSUC2:Avr1b (SP)-Invertase was used as a positive control. The YTK12 strain and YTK12 carrying the empty pSUC2 vector were used as negative controls. YTK12 can grow on YPDA plates. CMD-W media was used to ensure the expression of pSUC2-derived plasmids. Yeast transformants were grown on the YPRAA media to detect invertase secretion. Meanwhile, enzymic activity of invertase was further confirmed by the reduction of the dye 2, 3, 5-triphenyltetrazolium chloride (TTC) to the insoluble red-colored triphenylformazan. The experiment was repeated three times independently.

The 20-aa N-terminal sequence of Mg-PEL1, i.e., the 18-aa predicted SP sequence along with the subsequent two amino acids, was cloned into the yeast vector pSUC2. The yeast YTK12 carrying pSUC2:Mg-PEL1(SP)-Invertase and the positive control pSUC2:Avr1b (SP)-Invertase can grow on CMD-W and YPRAA media plates (Figure 3). Invertase enzymatic activity was further confirmed by the reduction of the dye TTC to the insoluble red colored triphenylformazan (Figure 3). By contrast, negative controls, i.e., YTK12 strain carrying pSUC2 empty vector and YTK12 strain, were not able to grow on YPRAA media, and culture filtrates were treated with TTC remained colorless (Figure 3). These results suggested that Mg-PEL1 carries a functional secretory SP.

### Developmental Expression Analysis

**FIGURE 4:**
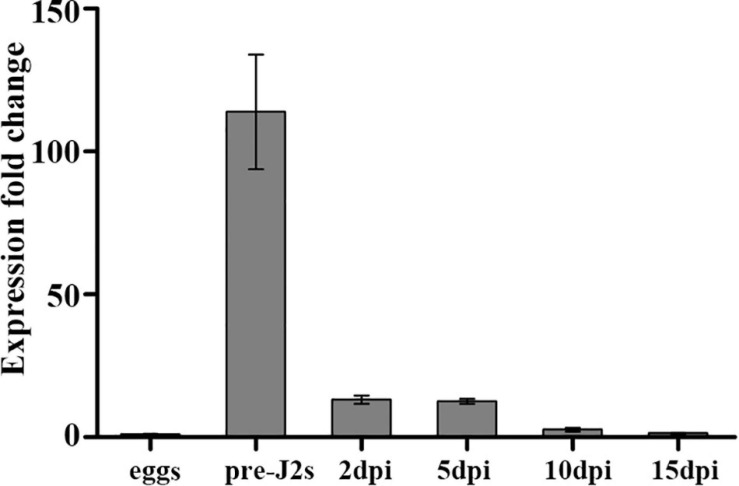
Expression of *Mg-PEL1* in *Meloidogyne graminicola* at different life stages. The fold change values were presented as the change in expression relative to the egg. Data shown are the means of three repeats plus standard deviation (± SD). Three independent experiments were performed with similar results. dpi, day post-infection; pre-J2s, pre-parasitic second-stage juveniles.

To analyze the developmental expression pattern of *Mg-PEL1*, qRT-PCR was performed by using RNA isolated from different developmental stages of *M. graminicola*. Transcripts at the egg stage was set unity as a reference to calculate the relative fold changes in other stages. The highest transcription level of *Mg-PEL1* appeared at the pre-J2 stage, representing 112-fold increases in expression compared with eggs. After the pre-J2 stage, the transcript level of *Mg-PEL1* declined dramatically. However, the relative fold changes for transcripts in parasitic nematodes at 2 and 5 day post-inoculation (dpi) still reached approximately 13-fold compared that in eggs (Figure 4). The findings suggested that Mg-PEL1 may play a role in the invasion and early migration stages of *M. graminicola*, especially in the invasion stage.

### Mg-PEL1 Exhibits a Hydrolytic Activity Toward PGA

**FIGURE 5:**
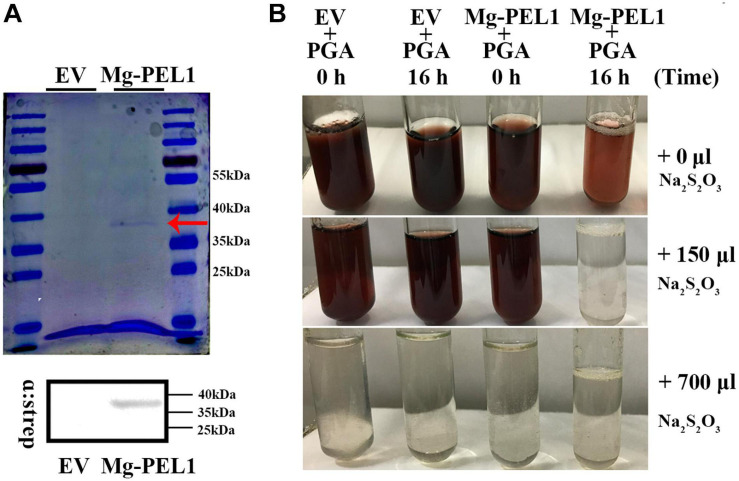
Enzyme activity analysis of recombinant protein Mg-PEL1. **(A)** Expression and purification of recombinant Mg-PEL1: Strep. Mg-PEL1: Strep was purified by anti-Strep beads and separated by SDS PAGE with Coomassie brilliant blue staining (red arrow). Western blot was used to confirm the correct expression of recombinant protein Mg-PEL1: Strep in culture supernatants. **(B)** Enzyme activity of Mg-PEL1 was measured using a titrimetric stop reaction assay. Mg-PEL1 possesses enzyme activity to hydrolyze PGA into oligogalacturonic acids, which can bind to free iodine. The rest amount of free iodine was titrated against Na_2_S_2_O_3_ using soluble starch as the indicator. The experiment was repeated three times. Similar results were obtained from three independent experiments. EV, empty vector; PGA, polygalacturonic acid.

To investigate enzymatic activity of Mg-PEL1, the yeast system was used to express the recombinant Mg-PEL1: Strep construct and empty vector (EV). Culture supernatants were purified using an anti-Strep bead and verified by Coomassie blue staining. A band at approximately 36 kDa molecular weight was observed in the Mg-PEL1: Strep construct culture supernatant, which corresponds to the predicted molecular weight of Mg-PEL1: Strep fusion protein. Western blot further confirmed the expression of Mg-PEL1: Strep. As a control, no bands were observed in the EV sample (Figure 5A), showing the correct expression of recombinant protein Mg-PEL1: Strep. For enzymatic activity analysis, culture supernatants of Mg-PEL1: Strep protein and EV were purified and incubated with free iodine and PGA solution for 0 and 16 h, respectively. The rest amount of free iodine was titrated against Na_2_S_2_O_3_ using soluble starch as the indicator. Titration results showed that the free iodine in the mixture containing Mg-PEL1: Strep protein at 16 h after incubation was lower than in other mixtures (Figure 5B). These results indicated that Mg-PEL1:Strep fusion protein has hydrolytic activity and can degrade PGA into oligogalacturonic acids, which are covalently bound to free iodine.

### Mg-PEL1 Is a Virulence Factor During *M. graminicola* Infection

**FIGURE 6:**
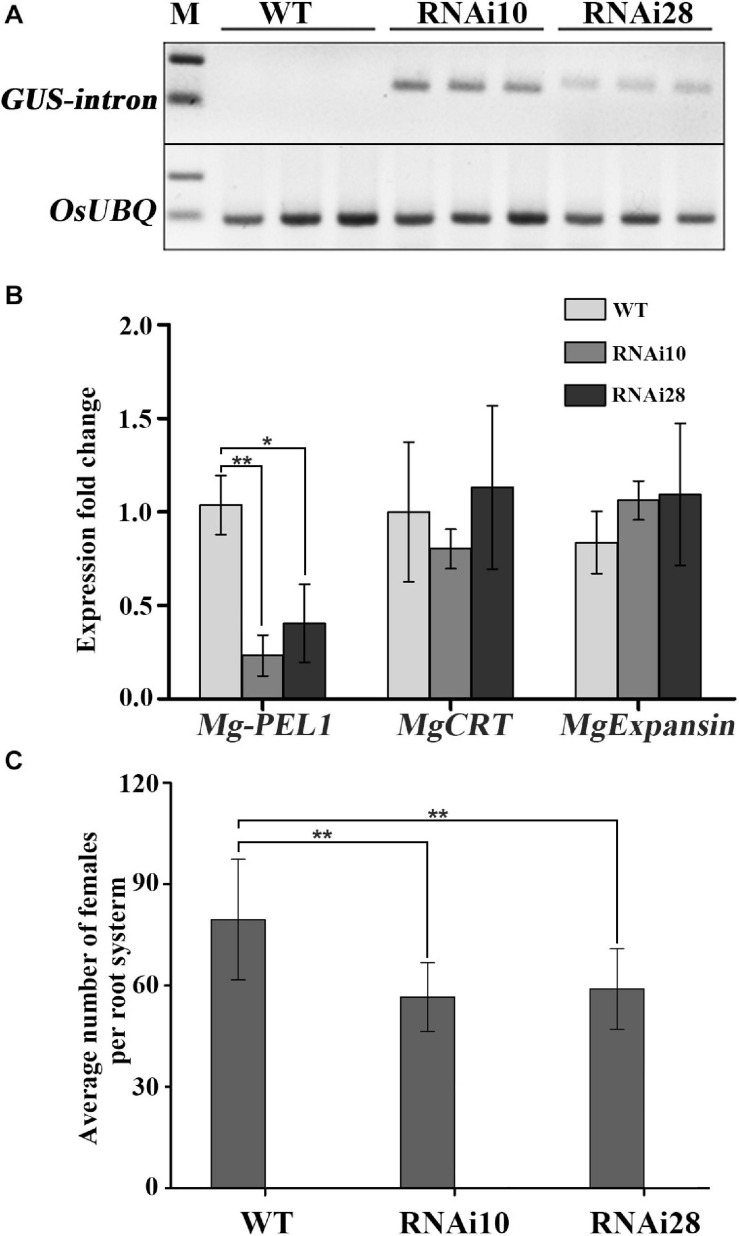
In planta RNA interference (RNAi) of *Mg-PEL1* reduces pathogenicity of *Meloidogyne graminicola*. **(A)** Semi-quantitative PCR for the detection of the β-glucuronidase (GUS) intron fragment was used to confirm the expression levels of RNAi construct in roots of transgenic RNAi lines. Three independent samples from different plants of WT, RNAi10 and RNAi28 were used for the analysis. The experiment was repeated three times. *OsUBQ* (Os03g13170) gene was selected as the reference gene for semi-quantitative PCR. **(B)** qRT-PCR assays of the expression levels of *Mg-PEL1* in *M. graminicola* collected from RNAi lines and WT at 3 day post-inoculation (dpi). The expression levels of *MgCRT* and *MgExpansin* from *M. graminicola* were used to determine the specificity of the *Mg-PEL1*-targeting RNAi. Similar results were obtained from three independent experiments. **(C)** Transgenic RNAi lines showed a significant reduction in the number of adult females compared with the WT. Two-week-old seedlings were inoculated with *M. graminicola* pre-J2s, and the number of adult females per plant was counted at 15 dpi. Data are presented as the means ± standard deviation (SD) from 9 to 10 plants. Two independent experiments were performed with similar results. All data revealed normal distribution and homogeneity of variance. One-way ANOVA Dunnett’s *t*-tests, *P < 0.05; **P < 0.01. RNAi10 and 28, different RNAi transgenic rice lines. WT, wild type.

To demonstrate the contribution of *Mg-PEL1* on *M. graminicola* virulence, host-mediated artificial double strands RNA (dsRNA) approach was utilized to silence the target gene *Mg-PEL1* within the feeding nematodes. The sense and antisense fragments of 368–703 bp within *Mg-PEL1* were separated by a GUS intron to construct a hairpin dsRNA vector targeting *Mg-PEL1* (Supplementary Figure 5), and the vector was transformed into rice. A 363-bp GUS intron fragment was detected by semi-qRT-PCR in two transgenic rice lines (#10 and #28), suggesting that these two lines may express *Mg-PEL1^368–703^* dsRNA. As control, no GUS fragments were amplified from WT plants (Figure 6A). The transgenic rice lines showed no significant differences compared with the WT plants (Supplementary Figure 6). To demonstrate the RNAi effect, qRT-PCR was used to detect the expression level of *Mg-PEL1* in nematodes at 3 dpi. The results showed that the transcription of *Mg-PEL1* in nematodes from RNAi lines was significantly reduced compared with that from the WT plants (Figure 6B). Because no similar sequences of *Mg-PEL1* were found in the genome and transcriptome of *M. graminicola*, two other *M. graminicola* effector genes *MgCRT* and *MgExpansin*, which have similar transcriptional expression patterns to *Mg-PEL1* were used to verify the specificity of this *Mg-PEL1*-targeting RNAi. qRT-PCR showed that these two non-targeting genes were not affected by this *Mg-PEL1*-targeting RNAi (Figure 6B). These results suggested that this *Mg-PEL1*-targeting RNAi was effective and specific. Meanwhile, RNAi lines, and WT plants were used for nematode infection assays. PCR was used to confirm the positive RNAi transgenic rice seedlings prior to nematode inoculation (Supplementary Figure 7). The results of infection assays indicated that two RNAi lines displayed a significant reduction in the number of adult females per root system compared with the WT plants at 15 dpi with *M. graminicola*. The number of adult females were found to be reduced by 22.6–25.7% (Figure 6C). These results suggested that *Mg-PEL1* affects *M. graminicola* parasitism.

### Cell Wall Localization of Mg-PEL1 Is Required for Activation of Plant Defense Responses

**FIGURE 7:**
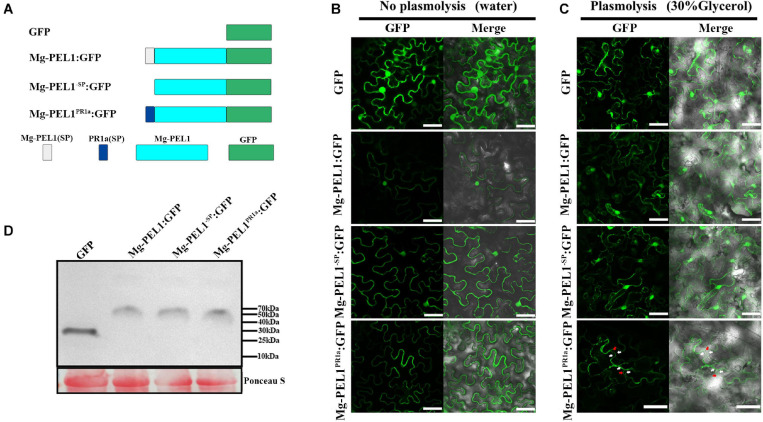
Subcellular localization of Mg-PEL1 in plant cells. **(A)** Schematic diagram showing the protein structures Mg-PEL1:GFP, Mg-PEL1^– SP^:GFP, Mg-PEL1^PR1a^:GFP and GFP. **(B)** *Agrobacterium* stain GV3101 carrying fusion constructs Mg-PEL1: GFP, Mg-PEL1^– SP^: GFP, Mg-PEL1^PR1a^:GFP and the GFP control were transiently expressed in *Nicotiana benthamiana* leaves. **(C)** *N. benthamiana* leaves expressing Mg-PEL1: GFP, Mg-PEL1^– SP^: GFP, Mg-PEL1^PR1a^: GFP and GFP were treated with 30% glycerol for plasmolysis. GFP signals were observed at 2 d after infiltration. Red arrows indicate plant cell wall, and white arrows indicate plasma membrane. Scale bar = 100μm. **(D)** Western blotting confirmed the expression of Mg-PEL1: GFP, Mg-PEL1^– SP^: GFP, Mg-PEL1^PR1a^: GFP and GFP in *N. benthamiana* leaves. Ponceau S staining was used to show equal loading.

Pectate lyases from pathogens usually target to plant cell wall and degrade plant cell wall polysaccharides (Hugouvieux-Cotte-Pattat et al., 2014). To investigate whether Mg-PEL1 can function in plant cell wall, three constructs Mg-PEL1^–SP^:GFP, Mg-PEL1:GFP and Mg-PEL1^PR1a^:GFP were first generated and transiently expressed in *N. benthamiana* leaves (Figure 7A). At 2 d post-infiltration, Mg-PEL1: GFP and Mg-PEL1^–SP^: GFP fusion proteins were observed in the cytoplasm and nucleus, while the Mg-PEL1^PR1a^: GFP fusion protein was accumulated in the edge of tobacco cells (Figure 7B). Plasmolysis was further performed to distinguish the plasma membrane from the cell wall. After the plasmolysis treatment, Mg-PEL1: GFP and Mg-PEL1^–SP^:GFP fusion proteins were still observed in the cytoplasm and nucleus, no signals observed in the extracellular space between plasma membrane and cell wall (Figure 7C). However, the Mg-PEL1^*PR*1a^: GFP fusion protein was found to localize in the cell wall and cytoplasm (Figure 7C and Supplementary Figure 8). Western blot performed on two dpi infiltrated leaves further confirmed the correct expression of these fusion proteins in tobacco cells (Figure 7D).

**FIGURE 8:**
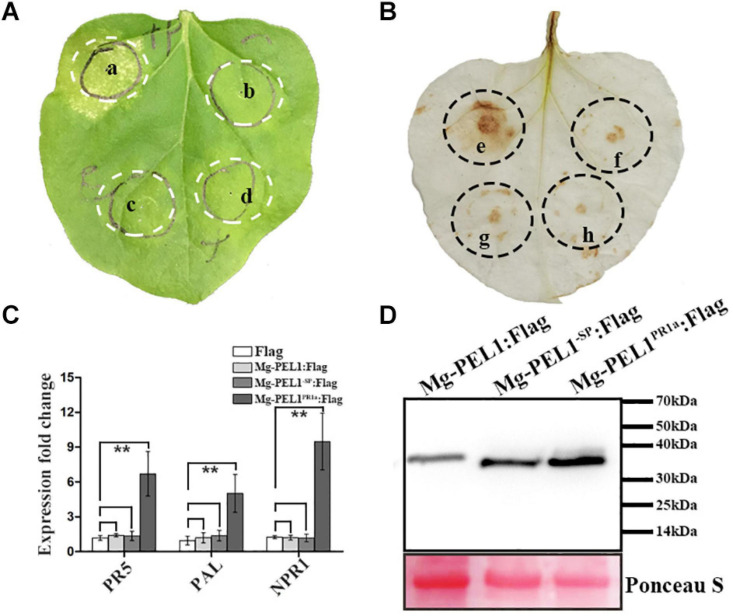
Cell wall localization of Mg-PEL1 is required for the activation of plant defenses. **(A)** Mg-PEL1 triggered cell death in *Nicotiana benthamiana*. *Agrobacterium* strain GV3101 carrying constructs Mg-PEL1^PR1a^: Flag (a), Mg-PEL1^– SP^: Flag (b), Mg-PEL1: Flag (c) and Flag (d) were transiently expressed in *N. benthamiana* leaves. At 5 d after infiltration, infiltrated sites were taken photos for cell death phenotype analysis. **(B)** Mg-PEL1 induced hydrogen peroxide production in *N. benthamiana*. *Agrobacterium* strain GV3101 carrying constructs Mg-PEL1^PR1a^: Flag (e), Mg-PEL1^– SP^: Flag (f), Mg-PEL1: Flag (g) and Flag (h) were transiently expressed in *N. benthamiana* leaves. At 2 d after infiltration, hydrogen peroxide production in infiltrated leaves was detected by DAB staining. **(C)** Mg-PEL1 induced the expression of defense related genes in *N. benthamiana*. The transcript levels of three defense response genes *PR-5*, *PAL* and *NPR1* were measured in *N. benthamiana* leaves at 2 d after infiltration with Mg-PEL1^– SP^:Flag, Mg-PEL1:Flag, Mg-PEL1^PR1a^:Flag and Flag constructs. All data revealed normal distribution and homogeneity of variance. One-way ANOVA Dunnett’s *t*-tests, *P < 0.05; **P < 0.01. **(D)** Western blotting confirmed the expression of Mg-PEL1^– SP^: Flag, Mg-PEL1: Flag and Mg-PEL1^PR1a^: Flag in *N. benthamiana* leaves. Ponceau S staining were used to show equal loading.

To investigate whether Mg-PEL1 can induce the defense responses when it accumulates in plant cell wall, four constructs Mg-PEL1^–SP^:Flag, Mg-PEL1:Flag, Mg-PEL1^PR1a^:Flag and Flag were then generated and transiently expressed in *N. benthamiana* leaves. First, the ability of Mg-PEL1 in induction of plant cell death was investigated. At 5 d post-infiltration, 23 of 30 infiltrated sites of Mg-PEL1^PR1a^: Flag displayed necrosis, whereas sites infiltrated with Mg-PEL1^–SP^: Flag, Mg-PEL1: Flag and Flag did not induce necrosis (Figure 8A). Second, the ability of Mg-PEL1 in hydrogen peroxide induction was detected. The DAB straining result showed that light-brown color was visible in the infiltration areas expressing Mg-PEL1^PR1a^: Flag, but invisible in infiltration regions with Mg-PEL1^–SP^: Flag, Mg-PEL1: Flag and Flag (Figure 8B). The results indicated that the expression of Mg-PEL1^PR1a^: Flag markedly promoted hydrogen peroxide production. Third, transcriptional levels of defense-related genes including *PR-5*, *PAL*, and *NPR1* were examined. Semi-qRT-PCR showed that the transcript abundances of the three defense-related genes were significantly up-regulated in *N. benthamiana* leaves expressing Mg-PEL1^PR1a^: Flag when compared to those in *N. benthamiana* leaves expressing Mg-PEL1^–SP^: Flag, Mg-PEL1: Flag and Flag (Figure 8C). The expression of all proteins was verified by western blot (Figure 8D). These results illustrated that Mg-PEL1 can activate plant defense responses only when it targets to plant cell wall.

## Discussion

In this study, we cloned and characterized a pectate lyase gene *Mg-PEL1* from the rice root-knot nematode, *M. graminicola*. Mg-PEL1 contains four highly conserved regions as well as five conserved cysteine residues, according to the class III pectate lyase (Shevchik et al., 1997). When compared with all the pectate lyase genes of PPNs, we found Mg-PEL1 at amino acid level shares <60% identities with them. Intriguingly, the phylogenetic analysis revealed that Mg-PEL1 has a closer relationship to several pectate lyases from fungi and bacteria than to some pectate lyases from PPNs, which is in line with the general idea that PPNs got pectate lyases from fungi or bacteria through horizontal gene transfer (Vanholme et al., 2007). These findings suggest that Mg-PEL1 may be a novel member of the class III pectate lyase.

Plant cell wall presents a primary defensive barrier against plant pathogens (Kubicek et al., 2014). As one of the important plant pathogens, PPNs can secrete various CWDEs to macerate the plant cell wall, which is essential during nematode invasion into plants or migration in plants (Kudla et al., 2005). Among these CWDEs, pectate lyases play a major role in the degradation of plant pectin, which is one of the most abundant components in plant cell wall (Caffall and Mohnen, 2009). Pectate lyases are widely distributed in diverse families of microorganisms and are considered as an important pathogenicity or virulence factor (Payasi et al., 2009). Recently, evidence emerged that PPN pectate lyases can promote nematode infection. For example, silencing of pectate lyase genes *Me-PEL1*, *Hs-PEL2*, and *Hg-PEL6* significantly reduced the ability of nematodes to infect host plants (Vanholme et al., 2007; Zhuo et al., 2011; Peng et al., 2016). In the present study, we showed that Mg-PEL1 has hydrolytic activity against PGA, a major component of pectin. Meanwhile, we observed that there was a significant decrease in the number of females in transgenic rice roots expressing the hairpin dsRNA vector targeting *Mg-PEL1.* These results demonstrated that Mg-PEL1 could be a virulence factor attacking the pectin of plant cell wall to promote nematode infection.

As a pathogenicity or virulence factor attacking plant cell walls, pectate lyases are usually secreted into the plant apoplast via the secretion system of pathogens (Blackman et al., 2014). For example, using immunolocalization analysis, *M. incognita*-derived pectate lyase Mi-pel3 was found to be localized within the subventral esophageal glands and secreted in the plant apoplast during root invasion of nematodes (Vieira et al., 2011). In PPNs, one of the most important secretory organs is the pharyngeal glands, which are composed of two subventral gland cells and one dorsal gland cell (Haegeman et al., 2012). The subventral glands are more active during nematode penetration and migration in roots (Davis et al., 2008). In our study, *Mg-PEL1* is specifically expressed in the subventral esophageal gland cells of *M. graminicola* by *in situ* hybridization. Additionally, the developmental expression profile of *Mg-PEL1* showed that the highest expression level occurred in the pre-J2 stage, and high expression level persisted in the par-J2 stage. Therefore, combining with the fact of Mg-PEL1 with an N-terminal SP, *Mg-PEL1* may probably be secreted during nematode invasion and migration in the roots, therefore playing a role in the penetration and migration of J2 in the host plant root, especially in penetration. It is believed that effector proteins with SP are cotranslationally transported into the endoplasmic reticulum of nematode esophageal gland cells, in which the SP is cleaved and the mature proteins are delivered into host tissue through the stylet of nematode (Elling et al., 2007). This is in line with our experiment that confirms the secretion function of the Mg-PEL1 SP using a yeast trap system.

In Eukaryotes, hydrophobic character of secretion signals is conserved and can be divided into three regions: a positively charge near the N-terminal end (n-region), a hydrophobic stretch of amino acids (h-region) and a polar C-terminal region leading up to the signal peptide cleavage site (Emanuelsson et al., 2000). The hydrophobic character of SP could be conserved between plant and animal (Schaaf et al., 2005). Several native SP containing-effector proteins from root-knot nematodes, including *M. incognita*-derived effector Mi-CRT, *M. graminicola*-derived effectors Mg16820 and Mg01965, have also been found to localize in the apoplast of host plant cells when transiently expressed (Jaouannet et al., 2013; Naalden et al., 2018a; Zhuo et al., 2019). However, several other factors besides hydrophobicity can also affect the complex interaction between SP and signal recognition particle, plants and animals are not exactly interchangeable (Matoba and Ogrydziak, 1998; Schaaf et al., 2005), raising an interesting question of whether the SP of Mg-PEL1 can be recognized by plants. Therefore, we used an *Agrobacterium*-mediated transient expression system to produce Mg-PEL1 with and without its native SP (Mg-PEL1^SP^ and Mg-PEL1^–SP^) in *N. benthamiana*. The results showed that they were present both in the cytoplasm and nucleus. This demonstrates that plants may not be able to perceive the native SP of Mg-PEL1 and Mg-PEL1^SP^ could not be transported to the plant apoplast.

The extracellular (apoplast) as a critical battleground for plants-microbes interaction is composed of intercellular space and plant cell wall (Ma Z. et al., 2017). Pathogen-secreted CWDEs including pectate lyases usually localize in the apoplast and degrade plant cell wall (D’Ovidio et al., 2004; Ma Y. et al., 2015; Gui et al., 2017). To deliver Mg-PEL1 into plant extracellular space, the SP of Mg-PEL1 was replaced with an endogenous SP sequence of PR1a (Mg-PEL1^PR1a^) from *N. benthamiana*. Subcellular localization showed that Mg-PEL1^PR1a^ can be accumulated at tobacco cells edge. Plasmolysis experiments also indicated that the Mg-PEL1^PR1a^ can target to the cell wall. Interestingly, Mg-PEL1^PR1a^ was capable of triggering host immune responses, including the induction of cell death, activation of defense-related genes and accumulation of hydrogen peroxide. However, neither Mg-PEL1 nor Mg-PEL1^–SP^ can induce host immunity. Plant innate immune system has been known to monitor the apoplastic environment through pattern recognition receptors that can detect fragments of host-derived molecules (DAMPs) as inducers of immune responses (Quintana-rodriguez et al., 2018). For instance, oligogalacturonides (OGs), a well-known fragment of the pectic polysaccharide homogalacturonan, were recognized as DAMPs by the receptor Wall Associated Kinase 1 to activate plant immune responses (De Lorenzo et al., 2011; Ferrari et al., 2013). Pectate lyases from *Xanthomonas campestris* pv. campestris with enzyme activity hydrolyzed plant pectate and produced OGs, which were subsequently recognized as a DAMP by the plant (Vorholter et al., 2012). In PPN-plant interaction, *Arabidopsis* polygalacturonase-inhibiting proteins (PGIP) mediated damage-associated responses during cyst nematode invasion of roots (Shah et al., 2017). PPNs secrete cell wall degrading enzymes to release cellular components that act as self-danger molecules known as DAMPs (Kaloshian and Teixeira, 2019; Sato et al., 2019). Combining with the results of Mg-PEL1 with hydrolytic activity toward PGA and immune activation ability relying on cell wall localization, Mg-PEL1 is likely to mediate the degradation of pectin in the plant cell wall, releasing pectin hydrolysis products to trigger defense responses in plants. In contrast, few studies such as HrpW protein belonging to pectin lyases 3 family from *Pseudomonas syringae* pv. tomato (Charkowski et al., 1998) showed that pectate lyases directly involved in plant immunity elicitation. As such, the possibility of plant immunity elicitation directly by Mg-PEL1 cannot be excluded. In PPNs, effectors GpPDI1 from *G. pallida*, MgPDI2 from *M. graminicola* and MG599854 from *M. arenaria* have been reported to induce cell death in the plant (Grijalva-Mañay et al., 2019; Gross et al., 2020; Tian et al., 2020). Recent studies also showed that pectate lyases have different functions in PPNs. Such as, silencing of *Hg-pel-2* that changed the sexual rate in favor of males (Bakhetia et al., 2007). *Gr-pel2* transient expression in *Nicotiana benthamiana* leaves led to severe malformations phenotype (Kudla et al., 2007). Either way, our study demonstrated the first example of PPN pectate lyases experimentally to trigger plant immune responses, although it has been shown previously that oomycete, fungal and bacterial pectate lyases play a role in plant immunity elicitation (Wegener et al., 1996; Poinssot et al., 2003; Fu et al., 2015; Yang et al., 2018).

Despite the ability of Mg-PEL1 to induce plant cell death, conversely, it also acts as an essential virulence factor during *M. graminicola* infection. It seems to be contradictory with the fact that root-knot nematodes are obligate biotrophic parasites and require living plant cells. Effector proteins with the ability to induce cell death are not preferred as a virulence factor for nematode parasitism. The similar contradictory phenomenon was also observed in several plant-pathogenic oomycetes effectors, such as PsXEG1 from *Phytophthora sojae*, Avh238 from *P. essential*, PpE4 from *P. parasitica* and PlAvh142 from *Peronophythora litchii* (Ma Z. et al., 2015; Yang et al., 2017; Huang et al., 2019; Situ et al., 2020). Two hypotheses have been raised to explain this. First, the accumulation of effectors or host-derived productions is insufficient to induce plant immune responses under natural conditions. Second, plant immune responses activated by effectors or host-derived productions could be suppressed by other effectors during successful parasitism of pathogens. As a typical example, the immune responses induced by the glycoside hydrolase family 12 protein PsXEG1 from *P. sojae* can be in turn suppressed by other RXLR effectors (Ma Z. et al., 2015). It will be interesting to determine whether *M. graminicola* can produce specific effectors to inhibit plant immunity induced by Mg-PEL1. And further studies on the exact mechanisms of plant immunity elicitation by Mg-PEL1 are also needed.

## Data Availability Statement

The datasets presented in this study can be found in online repositories. The names of the repository/repositories and accession number(s) can be found in the article/Supplementary Material.

## Author Contributions

KZ, JC, and BL: conceived and designed the experiments. JC and ZL: performed the experiments and data analysis. KZ, JC, and JL: manuscript preparation and submission. KZ, JC, and ZL: data curation and investigation. JC and BL: methodology. All authors contributed to the article and approved the submitted version.

## Conflict of Interest

The authors declare that the research was conducted in the absence of any commercial or financial relationships that could be construed as a potential conflict of interest.

## Footnotes

1 https://www.genome.jp/tools-bin/clustalw

2 http://sourceforge.net/projects/boxshade/

3 http://www.cbs.dtu.dk/services/SignalP/

4 http://www.cbs.dtu.dk/services/TMHMM/
