# Supplementary material for: A Meloidogyne graminicola Pectate Lyase Is Involved in Virulence and Activation of Host Defense Responses
Source: Front Plant Sci. 2021 Mar 22;12:651627. doi: 10.3389/fpls.2021.651627 (PMC8044864; doi:10.3389/fpls.2021.651627)
Supplement: Supplementary file 1 [file Data_Sheet_1.docx]

Supplementary Material

**Supplementary Table 1.** Primers used in this study.

| Primer | Sequence 5'- 3' | Purpose |
| --- | --- | --- |
| Mg-PEL1_F | ATGGCTTTTAATAATTTCATT | *Mg-PEL1* genomic fragment PCR amplification |
| Mg-PEL1_ R | TTACTTTCCACTTTTTTTCT |  |
| Mg-PEL1_insitu_F | GTCAAAGCAGATTTTTGGCC | *In situ* hybridization assays in *M. graminicola* |
| Mg-PEL1_insitu_R | TTTCCACAACAGCGAAGAAG |  |
| Mg-PEL1_qPCR_F | TGGTAAATCTGGCAATCCT | qRT-PCR for *Mg-PEL1* expression patterns in *M. graminicola* |
| Mg-PEL1_qPCR_R | CACAACAGCGAAGAAGAC |  |
| Mgactin2_F | ATGGCAACTGCCGCTTCTTCT | *M. graminicola* housekeeping gene (*ACT2*) used as qRT-PCR reference |
| Mgactin2_R | AGATTCCGGACAACGGAAGCGT |  |
| Mg-PEL1_EcoR1_F | AAA GAATTC ATGGCTTTTAATAATTTC | pSUC2:Mg-PEL1(SP)-invertase constructed for Mg-PEL1 signal peptide functional validation |
| Mg-PEL1_Xho1_R | AAA CTCGAG AAAATCTGCTTTGACAAA |  |
| Mg-PEL1_ fusion _F | TCCAAGCTCGGAATTTTAATTAAGAATTC ATGGCTTTTAATAATTTC | pSUC2:Mg-PEL1:Flag constructed for Mg-PEL1 fusion protein expression |
| Mg-PEL1_fusion_R | TTGACCAAACCTCTGGCGAATTACTATTTATCATCATCATC |  |
| GUS-qPCR_F | TTCTTGGTTAGGACCCTTT | qRT-PCR for GUS intron expression in RNAi lines |
| GUS-qPCR_R | AGTTCGTCGGTTCTGTAA |  |
| MgCRT_F | TTCTTCTCTTCCTCTTCA | qRT-PCR for *MgCRT* expression in *M. graminicola* affecting RNAi lines |
| MgCRT_R | GTGCTGTTGGTATTGATA |  |
| MgExpansin_F | CTCAGGCTCTTTATTTACATCA | qRT-PCR for *Mg-expansin* expression in *M. graminicola* affecting RNAi lines |
| MgExpansin_R | TTCGCATTCAGGACATTG |  |
| OsUBQ_F | CCAGTAAGTCCTCAGCCATGGAG | Rice housekeeping gene (OsUBQ) used as qRT-PCR reference |
| OsUBQ_R | GGACACAATGATTAGGGATC |  |
| Mg-PEL1^368-703^_sense_F | AAAGGATCCTATTTCAAATTGTATAAT | Mg-PEL1^368-703^constructed for RNAi assays in rice |
| Mg-PEL1^368-703^-sense_F | AAAAAGCTTAAATTGTCCAGGAGTTCC |  |
| Mg-PEL1^368-703^_antisense_F | AAAACGCGTTATTTCAAATTGTATAAT |  |
| Mg-PEL1^368-703^_antisense_R | AAACTGCAGAAATTGTCCAGGAGTTCC |  |
| PR1a_SP_F | AAA CCATGGGATTTGTTCTCTTTTC | PR1a signal peptide fragment amplification |
| PR1a_SP_R | TGACCTGGCACGGCAAGA |  |
| PR1a_Mg-PEL1_F | TCTTGCCGTGCCAGGTCAGATTTTTGGCCTGAAGCA | PR1a fusion with Mg-PEL1^-SP^ to construct Mg-PEL1^PR1a^: Flag |
| Mg-PEL1-PmlI_R | AAACACGTGCCTTTCCACTTTTTTTCT |  |
| Mg-PEL1_PR1a_BamHI_F | AAAGGATCCATGGGATTTGTTCTCTTTTC | PR1a fusion with Mg-PEL1^-SP^ to construct Mg-PEL1^PR1a^:GFP |
| Mg-PEL1_PstI_F | AAACTGCAGTTTCCACTTTTTTTCT |  |
| Mg-PEL1_BamHI_F | AAAGGATCCATGGCTTTTAATAATTTC | Mg-PEL1^-SP^:GFP and Mg-PEL1:GFP fusion constructs |
| Mg-PEL1_-SP_BamHI_F | AAAGGATCCGATTTTTGGCCTGAAGCA |  |
| Mg-PEL1_NcoI_F | AAA CCATGGATGGCTTTTAATAATTTC | Mg-PEL1^-SP^:Flag and Mg-PEL1:Flag fusion constructs |
| Mg-PEL1_-SP_ NcoI _F | AAA CCATGGGATTTTTGGCCTGAAGCA |  |
| PR-5_F | GGGCCAATCTTGGAGCATTA | Defense marker gene |
| PR-5_R | CAGTCTCCAGTCTCACAATTACC |  |
| NPR1_F | GGAGCAAGCAGAAAGAAGAGA | Defense marker gene |
| NPR1_R | GTTTAGCCAGGCCAACTCTAT |  |
| PAL_F | ATTGCTGGTTTGCTCACTGG | Defense marker gene |
| PAL_R | TCCTTAGGCTGCAACTCGAA |  |
| NbEF1α_F | AGTATGCCTGGGTGCTTGAC | *N. benthamiana* reference gene |
| NbEF1α_R | CAGGGACAGTTCCAATACCA |  |

ATGGGATTTGTTCTCTTTTCACAATTGCCTTCATTTCTTCTTGTCTCTACACTTCTCTTATTCCTAGTAATATCCCACTCTTGCCGTGCCAGGTCA

MGFVLFSQLPSFLLVSTLLLFLVISHSCRARS

**Supplementary Figure 1.** The signal peptide sequences of NbPR1a from *Nicotiana benthamiana*. The 32-aa N-terminal sequence of NbPR1a, i.e. the 30-aa predicted SP sequence plus the subsequent two amino acids

ATGGCTTTTAATAATTTCATTTTATTTTTTACATTTTTCTATTTTGTCAAAGCAGATTTTTGGCCTGAAGCAAGGCAGAATATAACG*gtaaatttttaatattttaattgtaattaaataaaaaaatttttaattttttcaatttgttccttttcttctatatgtaaaggatagagcacattccccttttcttctttaaaaaattaattttttttctttaaaaacag*CTTATCGAAACAAAAATAATTGATGGTGTCTTTGACTGTGAATATGATCGTTATATTCCTGATCCTAAAAAAATGGGAAATGGAGGTCAATCCGAAAATCAAAAAAGAGTTTTTGACTTAAATGACGGTGCAACTATTTCAAATTGTATAATTGGTGTAAAACCTGGTGCCATTGGACCAGCAGATGGAATTAGATGTTTGGGAAGTTGTACAATAAATAATGTTTGGTTTGAGACTGTTGGGGAAGATGCAATTACTTTTTATGGTAAATCTGGCAATCCTGTATATCATGTTAATGGTGGTGGTGCAAGACATGGAAAAGATAAAACCTTTCAATTTGATGGAAAAGGAACGACATACATTGATAATTATTAT*gtaagtataaataacaatgattaaaaatattttaaag*GTTGATGATTATGTACGTCTTCTTCGCTGTTGTGGAAACTGTCCAAACCAATTTCAACGTAATGTAGTAATTCGTAATTTAACTGCAATAAATGGAACTCCTGGACAATTTATTGTTGGAATAAATAAAAATTATGGAGACACTGCAAAATTAAGTCAAATAAAAATGGAAAATGGTGTACATCCTTGTAAGTTATTTACTGGTAATAATAATGGAGCTGAACCACAATCTATTGGAACTGAAGAAGATGGAAAATATTGTATTTATAATGAAGGAGATATTACTTATTTATCTACAACCAGCTCTAAAAAGAAGAAAAAAAGTGGAAAGTAA

MAFNNFILFFTFFYFVKADFWPEARQNITLIETKIIDGVFDCEYDRYIPDPKKMGNGGQSENQKRVFDLNDGATISNCIIGVKPGAIGPADGIRCLGSCTINNVWFETVGEDAITFYGKSGNPVYHVNGGGARHGKDKTFQFDGKGTTYIDNYYVDDYVRLLRCCGNCPNQFQRNVVIRNLTAINGTPGQFIVGINKNYGDTAKLSQIKMENGVHPCKLFTGNNNGAEPQSIGTEEDGKYCIYNEGDITYLSTTSSKKKKKSGK

**Supplementary Figure 2.** The gDNA sequence of *Mg-PEL1.* The predicted start codon and stop codon are in red; Two introns are presented in italic and lower-case letters; The predicted signal peptide is underlined.


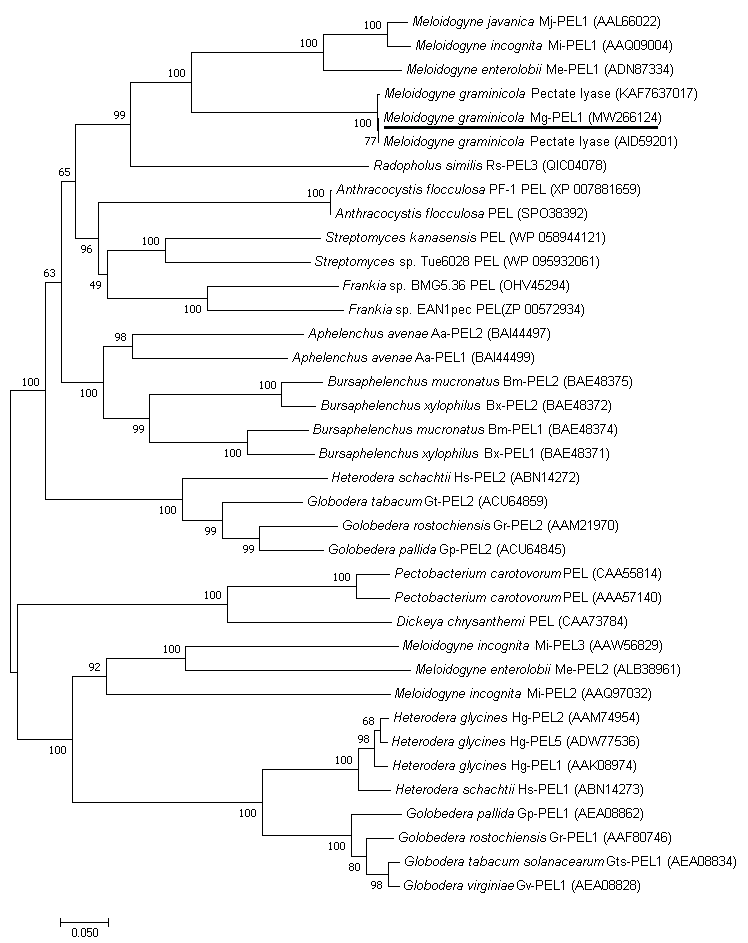


**Supplementary Figure 3.** Phylogenetic tree of the pectate lyases from plant-parasitic nematodes, fungi and bacteria.

**
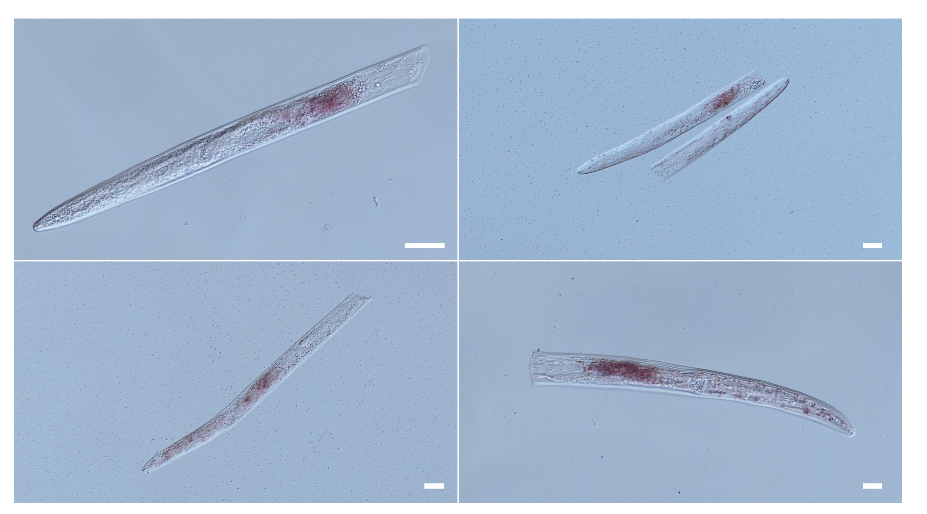
**

**Supplementary Figure 4.** Localization of *Mg-PEL1* in the subventral esophageal gland cells of pre-parasitic second-stage juveniles of *M. graminicola* with digoxigenin-labeled *Mg-PEL1* antisense cDNA probes. Scale bars, 20 μm.


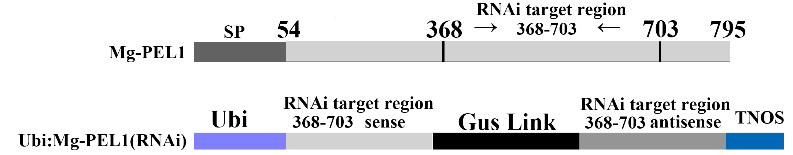


**Supplementary Figure 5.** The scheme of the RNA interference constructs used in rice transformation. Constructs generated for host-derived RNA interference (RNAi).


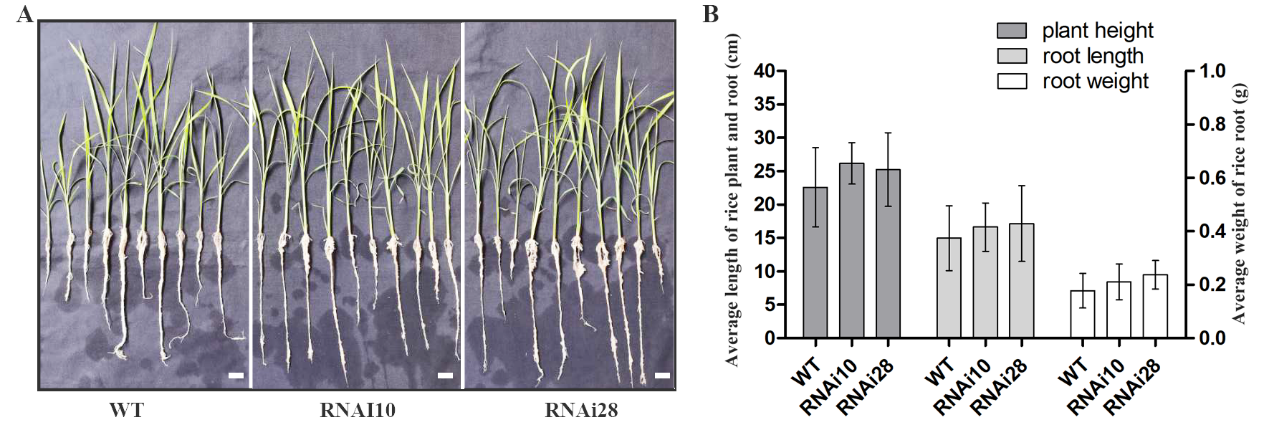


**Supplementary Figure 6.** Phenotype analysis of *Mg-PEL1* RNAi transgenic rice lines. Two independent transgenic rice lines (RNAi10 and RNAi28) and wild type (WT) seeds germinated on culture dishes for 3 days and then grown on quartz sand for 24 days. Comparison of the average length of *Mg-PEL1* RNAi transgenic rice lines with WT showed that transgenic lines did not display significant difference in plant height, root length and root weight. Data are presented as means ± standard deviation (SD) (n=10). *P < 0.05; **P < 0.01, student’s t test. RNAi10 and RNAi28, two transgenic rice lines. Scale bar, 2 cm.


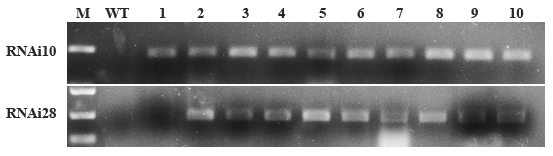


**Supplementary Figure 7.** Genomic PCR confirmation of *Mg-PEL1* RNAi transgenic lines. gDNA PCR was used to confirm the fragment of *Mg-PEL1^368-703^* in RNAi transgenic lines. RNAi10 and RNAi28, two transgenic rice lines; WT, wild type. 1-10, different transgenic rice plants. M, DNA marker. RNAi10 and RNAi28, two different transgenic rice lines.


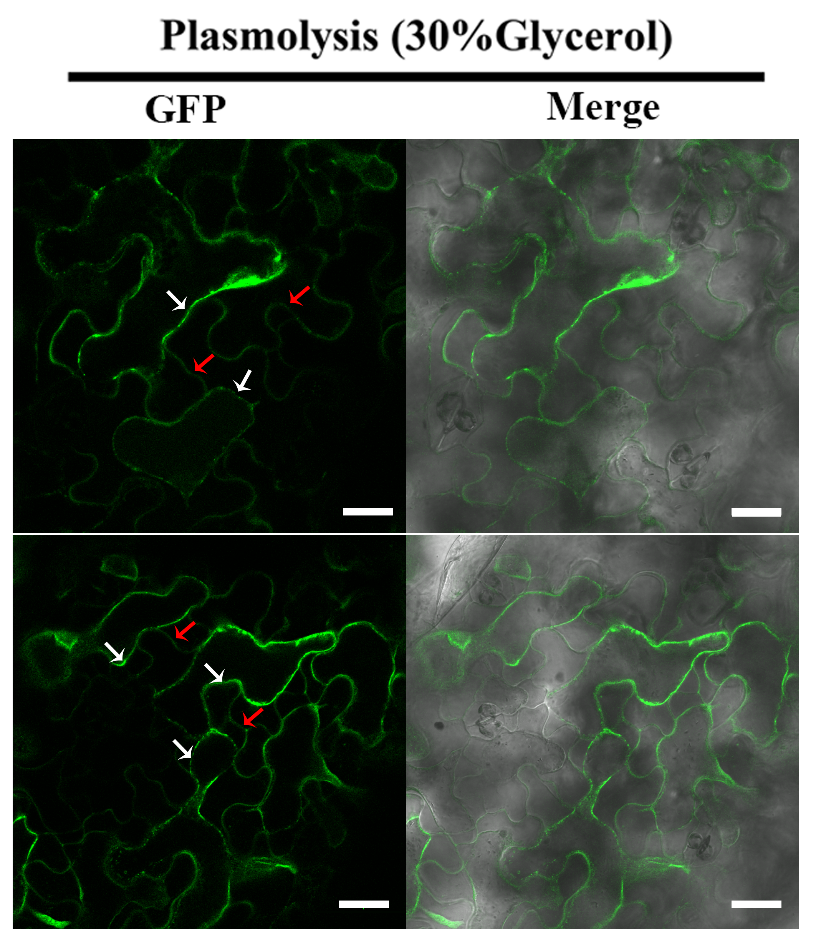


**Supplementary Figure 8.** Subcellular localization of Mg-PEL1 in the plant cells. *Agrobacterium* strain GV3101 carrying the fusion construct Mg-PEL1^PR1a^:GFP was transiently expressed in *Nicotiana benthamiana* leaves. 2 days after infiltration, *N. benthamiana* leaves were treated with 30% glycerol for plasmolysis. All leaves were observed under confocal microscopy. Red arrows indicate plant cell wall, and white arrows indicate plasma membrane. Scale bar =100 μm.
